# β-Antithrombin Levels in Patients with Venous Thromboembolism

**DOI:** 10.3390/ijms262211151

**Published:** 2025-11-18

**Authors:** Edith Alexandra Uj, Éva Molnár, Tünde Miklós, Réka Gindele, Amir Houshang Shemirani, Zsuzsanna Bereczky, Éva Katona

**Affiliations:** 1 Division of Clinical Laboratory Science, Department of Laboratory Medicine, Faculty of Medicine, University of Debrecen, Nagyerdei Blvd. 98, 4032 Debrecen, Hungary; uj.alexandra@med.unideb.hu (E.A.U.); molnare@med.unideb.hu (É.M.); gindele.reka@med.unideb.hu (R.G.); shemirani1@gmail.com (A.H.S.); zsbereczky@med.unideb.hu (Z.B.); 2 Kálmán Laki Doctoral School, University of Debrecen, 4032 Debrecen, Hungary

**Keywords:** clotting, antithrombin, β-antithrombin, thrombosis, venous thromboembolism

## Abstract

Beta-antithrombin (β-AT), the isoform of antithrombin (AT) with a higher affinity for heparin, constitutes 5–10% of total AT in plasma. There are limited data regarding β-AT activity levels in thrombotic disorders. In our study, we analyzed samples from 200 non-AT-deficient patients who had experienced venous thromboembolism (VTE) compared to 200 healthy controls. Total AT activity was measured using a chromogenic anti-factor Xa assay. To measure β-AT, we used elevated NaCl (1.1 M) in the reagent to inhibit the heparin binding of α-AT. There were no significant differences in total AT activity (median (IQR)) levels between the control and VTE groups (100 (93–109)% and 99 (94–109)%, respectively; *p* = 0.955). However, the β-AT activity levels (median (IQR)) and the ratio of β-AT to total AT (mean ± SD) were significantly higher in the VTE group compared to the control group (93.3 (90.3–97.3)% vs. 89.3 (84.0–95.0)% and 9.34 ± 0.68% vs. 8.86 ± 0.88%; *p* < 0.001). β-AT activity levels and the ratios in the upper third were strongly associated with a higher risk of VTE (OR (95% CI): 5.78 (3.08–10.87) and 6.15 (3.36–11.24), respectively). Our study demonstrated an elevation of plasma levels of β-AT in patients with VTE. Further research is necessary to clarify the pathophysiological significance of this finding.

## 1. Introduction

Antithrombin (AT) is a key regulator of hemostasis, being the primary inhibitor of coagulation. It is synthesised in the liver, has a molecular weight of 58 kDa, and has a concentration of 0.150 mg/mL (~2.6 μM) in the normal plasma [1]. It belongs to the serine protease inhibitor family (known as SERPINs), therefore has the highly conserved structural features characteristic of SERPINs [2,3,4].

AT inhibits not only activated factor II (thrombin), but also activated clotting factors as factor X (FXa), factor IX (FIXa) [5], factor XI (FXIa) [6], factor XII (FXIIa) [7], and factor VII (FVIIa) [8]. AT alone is a slow-acting and inefficient inhibitor. However, the binding of AT via its positively charged heparin-binding domain to negatively charged glycosaminoglycans (GAGs) like heparin or heparan sulfate accelerates the rate of inhibition by several hundred-fold [9,10]. AT is essential for the regulation of the coagulation cascade: its complete absence has been proven to be incompatible with life in animal studies [11,12].

AT has two isoforms, 90–95% of the circulating AT being α-antithrombin (α-AT) and 5–10% β-antithrombin (β-AT). The two forms differ from each other in their glycosylation pattern. While in α-AT all four potential sites are glycosylated, β-AT lacks the carbohydrate at Asn135 [13]. The β-isoform has a higher affinity for heparin [14]; therefore, it is considered to be a more potent inhibitor, being mainly responsible for the inhibition of thrombin on the subendothelial surface [15,16]. In addition, the β-AT also showed a higher inhibitory effect against in vitro induced endothelial damage [17].

The laboratory testing of AT primarily serves the purpose of diagnosing AT deficiency. The currently available laboratory methods are only suitable for measuring the total AT activity or antigen levels. The AT activity assays are chromogenic methods performed in the presence of heparin and based on the determination of residual thrombin or FXa activity after excess thrombin or FXa was added to the AT-containing sample [18]. The reference range of AT activity is typically 80–120%. Progressive activity is measured using the same principle, but in the absence of heparin, and its main scope is to differentiate between the different types of functional antithrombin deficiencies [19]. The total AT antigen concentration can be determined by immunological assays such as enzyme-linked immunosorbent assay (ELISA) [20].

To date, there is no existing commercially available diagnostic laboratory method for measuring β-AT levels in the plasma. However, several studies have used a modification of their AT activity assay (regardless of whether they were based on thrombin or FXa determination), in which the salt concentration is increased to inhibit the binding of α-AT to heparin [21,22,23].

Venous thromboembolism (VTE) represents a significant public health concern worldwide since it is a major contributor to preventable deaths [24]. It is a well-established fact that both hereditary and acquired AT deficiency are serious risk factors for developing VTE [25]. However, it appears that non-antithrombin-deficient individuals with AT activity levels in the lower part of the normal range also exhibit an elevated risk of experiencing a VTE episode [26]. AT activity levels between 70 and 80% of the normal average may be classified as mild AT deficiency. According to the findings of previously published studies, mild AT deficiency can lead to a 1.6–3.7-fold higher risk of recurrent VTE [27,28]. Additionally, the risk of VTE rises progressively with the decreasing AT activity levels within the normal range [29]. There are currently no data on β-AT levels in non-antithrombin-deficient patients with VTE. A single study investigated plasma levels of β-AT in individuals with ischemic cerebrovascular disease during the acute phase and again one year later. Both β-AT activity and the β/total-AT ratio were significantly elevated during the acute event but returned to baseline after one year. The observed elevation was hypothesized to result from the release of β-AT from the vasculature [22].

We hypothesized that endothelial dysfunction, which is a risk factor for VTE, affects the surface sequestration of β-AT, resulting in altered circulating β-AT activity; therefore, in this study, we aimed to investigate β-AT activity levels in patients who suffered VTE before compared to healthy control subjects by using a modified version of the routinely used chromogenic AT activity assay.

## 2. Results

### 2.1. Characterization of the Study Population

Table 1 shows the basic clinical and laboratory characteristics of the study population. There were no significant differences in the mean age or the distribution of males and females between the VTE group and the control group. The two groups did not significantly differ in their smoking habits and family history of VTE. Concerning genetic risk factors for developing VTE, the prevalence of both heterozygous and homozygous factor V Leiden mutation was significantly higher in the VTE group. However, there was no significant difference in the prevalence of the prothrombin 20210G>A mutation.

Out of the 200 patients with VTE, 152 had suffered deep vein thrombosis (DVT) only, while 42 patients had developed pulmonary embolism (PE) secondary to the DVT. In the case of 6 patients, only PE was identifiable. Among those diagnosed with DVT, 55% had distal thrombosis, while 41% had proximal thrombosis. In 4% of DVT patients, the thrombus affected the upper extremity. In the patient group, 76% of cases had no identifiable acquired risk factors. Among the remaining 24% of patients, the following provoking factors occurred: oral contraceptive or hormone therapy use (*n* = 13), trauma or immobilization (*n* = 12), surgery (*n* = 8), pregnancy or postpartum period (*n* = 8), lupus anticoagulant (*n* = 2), cancer (*n* = 3), and varicosity (*n* = 2).

The majority of patients with VTE (158 cases) received vitamin K antagonist treatment, 12 patients received heparin therapy, and 18 patients were on direct oral anticoagulation (DOAC) treatment, including thrombin or FXa inhibitors. The remaining 12 patients received no therapy at the time of blood sampling.

The median BMI and the mean levels of fibrinogen, CRP, total a2-PI antigen, and NPB-a2-PI antigen were higher in the VTE group compared to the control group. The prevalence of high blood pressure was significantly higher in the VTE group. There was no significant difference in total cholesterol, HDL cholesterol, and LDL cholesterol levels between the groups; however, the triglyceride levels were significantly lower in the patient group.

### 2.2. The Evaluation of β-AT Activity Assay

The β-AT activity assay has satisfactory reproducibility. The within-run and between-run coefficients of variation (CV) for normal as well as pathological control plasmas were 2.8% and 3.0%, and 4.0% and 4.7%, respectively.

**Figure 1:**
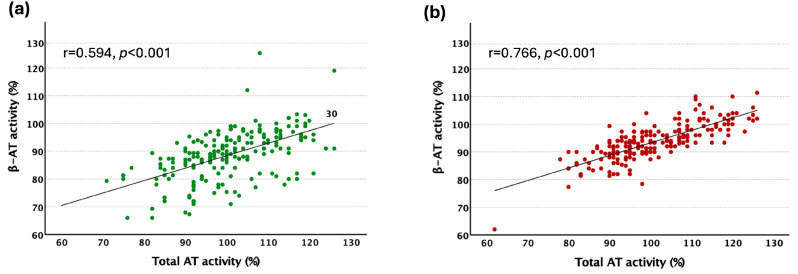
Correlation of total AT and β-AT activity measurements in controls; *n* = 200 (**a**) and in the patient; *n* = 200 (**b**) group. Values are expressed as Spearman’s rho, significance *p*.

β-AT activity and total AT activity levels showed a good positive correlation in both groups (Figure 1).

### 2.3. Total-AT and β-AT Levels in VTE Patients

**Figure 2:**
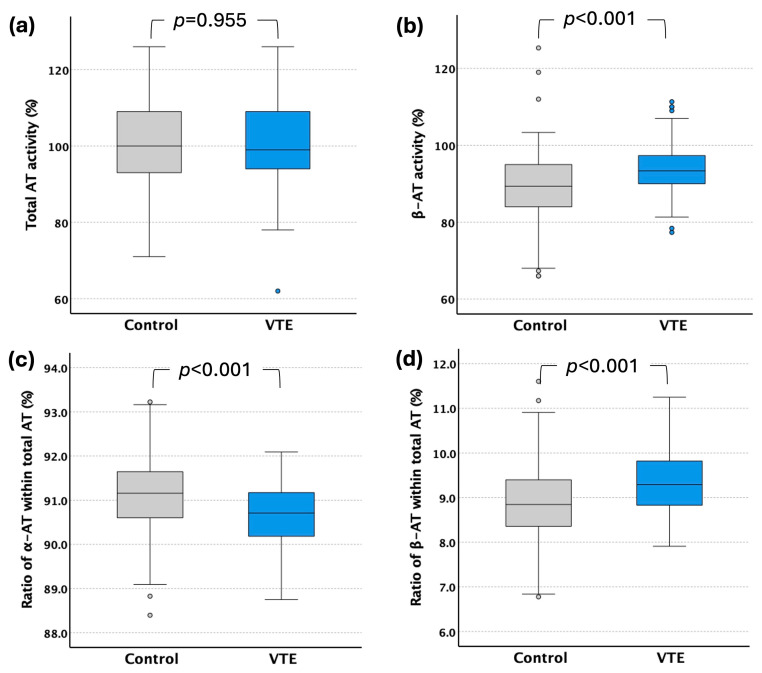
Plasma levels of different AT forms in control (*n* = 200) and VTE group (*n* = 200): (**a**) total AT activity, (**b**) β-AT activity, (**c**) ratio of α-AT within total AT (%), (**d**) ratio of β-AT within total AT (%). The results are presented as boxes (median and the intervals between 25th and 75th percentiles) and whiskers (2.5th and 97.5th percentiles); circles denote cases in the lower and upper 2.5th percentile.

Total AT activity levels did not show a statistically significant difference between controls and patients with VTE. In contrast, β-AT activity levels and the ratio of β-AT within total AT (%) elevated significantly in the VTE group. The ratio of α-AT within total AT (%) was lower in the patient group (Figure 2, Table 2). There were no sex-related differences in total AT activity levels and the ratio of β-AT within total AT (%); however, β-AT activity levels were significantly higher in women experiencing VTE (Table 2).

**Figure 3:**
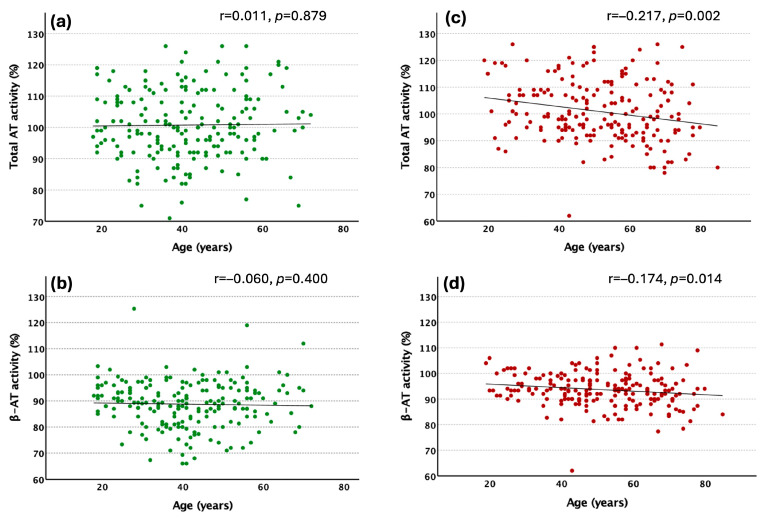
Correlation of different AT parameters with age in the control (*n* = 200) (**a**,**b**) and VTE group (*n* = 200) (**c**,**d**). Spearman’s rank correlation coefficients and the levels of statistical significance are shown on the figures.

Total AT and β-AT activity levels showed a weak negative correlation with age in patients (Figure 3c,d), but not in controls (Figure 3a,b). In the control group, using a multiple linear regression analysis, total AT activity showed an independent negative association only with the CRP levels (Standardized β = −0.226, *p* = 0.025), and the β-AT activity levels showed an association only with the total AT levels (Standardized β = 0.490, *p* < 0.001). In the VTE group, total AT showed an independent association with age (Standardized β = −0.261, *p* < 0.001), fibrinogen (Standardized β = 0.282, *p* = 0.022), CRP (Standardized β = −0.182, *p* = 0.043), and total cholesterol (Standardized β = 0.168, *p* = 0.017), while β-AT activity levels showed independent association with total AT (Standardized β = 0.744, *p* < 0.001), and total cholesterol (Standardized β = 0.200, *p* < 0.001).

### 2.4. β-AT Levels and Thrombotic Risk

A logistic regression analysis comparing cases with β-AT activity in the upper third with those in the lower third showed a strong association between β-AT activity and VTE. The same result was found for the ratio of β-AT within total AT (Table 3). Performing logistic regression analysis, by inserting all investigated variables as covariates, BMI, hypertension, FV Leiden mutation, FII 20210G>A mutation, CRP, and NPB-α2-PI antigen levels showed a significant association with the occurrence of VTE. OR values increased after adjustment for these thrombotic risk factors. Conversely, total AT activity levels did not show a significant association with the risk of VTE (Table 3). The results of logistic regression analyses treating total AT, β-AT, and β-AT/total AT ratio as continuous predictors are shown in the Supplementary Table S1.

## 3. Discussion

Only a few large-scale studies have investigated the relationship between total AT activity levels and the risk of VTE in non-antithrombin-deficient patients [26,27,28,29]. According to these studies, antithrombin activity levels in the lower part of the normal range are associated with an elevated risk of VTE. Our results failed to support these findings, most likely due to our smaller sample size.

To our knowledge, this is the first study to determine β-AT activity levels in patients with VTE. Because there are no commercially available methods to measure β-AT activity, there is limited data on how β-AT levels change in thrombotic disorders. Following the principle of the method published by de la Moreno-Barrio et al. and Karlaftis et al. [21,22], with the elevation of NaCl concentration of the reagents to 1.1 M, we modified our routinely used chromogenic total AT activity assay to inhibit the heparin cofactor activity of the α-AT and measure only the residual β-AT activity. This modified assay demonstrated good performance characteristics.

We found a positive correlation between β-AT activity levels and total AT activity levels (Figure 1) in the control and VTE groups. Our results do not confirm the findings of de la Morena-Barrio, who reported no significant correlation between total- and β-AT levels in 97 healthy subjects from the general population [22].

In our study, total- and β-AT activity levels did not correlate with age in the control group. This supports the findings of previous studies [21,22]. However, both total- and β-AT levels exhibited a weak negative association with age in the patient group, which relationship was independent of the effect of other factors. No difference in the total AT activity and the ratio of β-AT within total AT was observed between men and women in either study group, which is consistent with the previously reported data [22].

We found that β-AT activity levels and the ratio of β-AT within total AT (%) were significantly elevated in patients with VTE compared to controls. Both β-AT levels and the ratio of β-AT within total AT in the upper third were strongly associated with the risk of VTE (OR: 5.78 and 6.15, respectively). In our study, the median BMI levels and the mean fibrinogen and CRP levels were higher in the VTE group compared to the control group. These findings are consistent with prior research [30,31,32]. The prevalence of hypercholesterolemia and high blood pressure was significantly higher in the VTE group, in line with previous studies [33,34]. The mean levels of total a2-PI antigen and NPB-a2-PI antigen were also higher in the VTE group compared to controls, which has previously been shown to increase the risk of VTE [35]. As expected, the prevalence of both heterozygous and homozygous factor V Leiden mutation was significantly higher in the VTE group. The prevalence of the heterozygous factor V Leiden mutation in the control group (12.5%) corresponds to what has been described in the general Hungarian population [36]. After adjustment for these thrombotic risk factors, OR values increased (to 12.2 and 6.4, respectively), showing that the elevated β-AT level is independently associated with VTE. According to a previous study, β-AT activity levels rise during an acute ischemic stroke event but return to normal after 12 months [22]. This is a surprising finding, since it is unexpected that the level of a potent endogenous anticoagulant would rise during a thrombotic event. The authors supposed that the elevation of plasma β-AT level is caused by its release from the vasculature, leading to a decrease in the amount of β-AT bound to the subendothelium and therefore decreasing the anticoagulant capacity of the vasculature itself. In our study, sampling occurred at least 3 months after the acute event, indicating that in patients with venous thrombosis, elevated β-AT levels persist for a long time. Moreover, we found no correlation between plasma levels and the time elapsed between sampling and the acute event. A possible explanation for the observed elevation of β-AT levels in thrombotic states is the altered β-AT binding capability of the dysfunctional endothelium that results in more circulating β-AT in the plasma. Both AT isoforms are bound to glycosaminoglycans within the endothelial glycocalyx, but β-AT does so more effectively due to its higher heparin affinity [17]. It has been shown that inflammatory conditions lead to endothelial damage and the release of GAGs [37,38]. Since thrombotic disorders are often accompanied by inflammatory processes, it is plausible that, due to endothelial damage, the binding of β-AT to GAGs is negatively affected, resulting in elevated β-AT activity levels in the plasma. Further studies are needed to determine whether the increase in beta-AT activity in plasma is more pronounced in acute VTE and whether it may be suitable as a biomarker for indicating endothelial dysfunction.

The study has several limitations. One of them is that the blood sample collection occurred at least 3 months following the VTE event; therefore, we did not measure the investigated parameters in the acute phase of the disease. Moreover, we only measured beta-AT activity levels and did not confirm the results by determining antigen levels; however, currently, no method exists to quantify β-AT antigen levels. Despite the limitations, our study might initiate further studies investigating the correlation of beta-AT levels with the severity of thrombosis, and whether beta-AT levels are a good predictor of long-term outcomes, including the risk of recurrent thrombosis or inadequate response to anticoagulant therapy. If a larger study proves that elevated beta-AT is a good marker of endothelial dysfunction, it could be incorporated into cardiovascular risk assessment.

## 4. Materials and Methods

### 4.1. Subjects

Two hundred consecutive patients diagnosed with VTE who were admitted to the Thrombosis Centre of the University of Debrecen during a one-year period were enrolled in the study. Deep vein thrombosis (DVT) was confirmed by color Doppler ultrasonography or venography, and pulmonary embolism (PE) was diagnosed according to the guidelines of the European Society of Cardiology [39]. Blood samples were collected at least 3 months after the thrombotic event. The median (IQR) time elapsed between the thrombotic event and blood sampling was 3.6 (1.6–6.7) years. Patients with known congenital antithrombin deficiency or AT activity below 60% were excluded from the study.

An equal number of age-matched healthy individuals were selected for the control group. Exclusion criteria for the control group were a previous VTE episode, malignancy, type I or type II diabetes, pregnancy, and chronic diseases except for well-controlled high blood pressure.

The demographic data and relevant medical history of the subjects were collected through a questionnaire. Venous blood samples were collected during a standard venipuncture procedure into vacutainer tubes containing 3.2% sodium citrate (Greiner Bio-One, Kremsmünster, Austria). Platelet-poor plasma was obtained by centrifugation at 2890× *g* for 15 min. Samples were aliquoted, snap frozen, and stored at −80 °C until analysis.

The work was carried out according to the principles laid down in the Declaration of Helsinki. The study protocol was approved by the Institutional Ethics Committee of the University of Debrecen and the Ethics Committee of the National Medical Research Council of Hungary (ETT TUKEB: 54005-3/2016/EKU). The participants provided their written informed consent to participate in the study.

### 4.2. Heparin Cofactor Antithrombin Activity Assay

To measure the total AT activity in the plasma samples, a spectrophotometric heparin cofactor assay was performed (LX Antithrombin Hc + P (FXa), Labexpert Kft., Debrecen, Hungary) on a Siemens BCS coagulometer (Siemens Healthineers, Erlangen, Germany). During the assay, the sample is diluted 1:50 with a buffer (50 mmol/L Tris-HCl, 175 mmol/L NaCl, 7.5 mol/L EDTA, and 10 mg/L polybrene, pH 8.4) containing heparin, then FXa is added in excess. The residual uninhibited FXa is measured by adding a specific chromogenic substrate (BIOPHEN CS 11(32), HYPHEN BioMed, Paris, France) and detecting the released para-nitroaniline(pNA) kinetically at 405 nm for 60 s. The amount of the generated pNA is inversely proportional to the amount of AT in the plasma. The calibration curve was created using a dilution series of HemosIL™ Calibration plasma (Instrumentation Laboratory, Milano, Italy).

To make the assay specific for β-AT, sodium chloride (NaCl) was added to both the sample diluent buffer and the FXa reagent to get a final concentration of 1.1 M. This high NaCl concentration prevents the α-isoform of AT from binding to heparin due to its weaker heparin affinity, allowing for the detection of only the heparin cofactor activity of the β-isoform. Since β-AT constitutes about 10% of the total AT in normal plasma, a decreased sample dilution (1:5) is used during the β-AT activity assay. In total, 100% of total AT corresponds to a concentration of 150 mg/L–~2.6 μM—while 100% β-AT corresponds to a concentration of 15 mg/L–~0.26 μM [22]. For every sample, we measured the total heparin cofactor AT activity (%), the β-AT heparin cofactor activity (%), and calculated the concentrations and the ratio of β-AT within total AT. The plasma samples of patients receiving direct oral anticoagulant therapy (DOAC) were treated with DOAC-Stop tablets (Haematex, Hornsby, Australia) to eliminate the interference with the performed assays. All assays were performed using the BCS XP automated hemostasis analyzer (Siemens Healthineers, Erlangen, Germany).

### 4.3. Method Evaluation

For the evaluation of the precision performance of the β-AT activity assay, commercially available control plasmas (Control Plasma N and P, Siemens Healthineers, Erlangen, Germany) were used. To determine the within-run and between-run precisions, the control plasmas were measured 11 times in the same run and on 14 different days, respectively.

### 4.4. Other Laboratory Methods

Fibrinogen concentration was measured using the Clauss method on a Siemens BCS coagulometer (Siemens Healthineers, Erlangen, Germany). High-sensitive C-reactive protein (hsCRP), triglyceride, total cholesterol, HDL cholesterol, and LDL cholesterol levels were measured by routine laboratory methods on Roche/Hitachi Modular P800 Analytics System (Roche, Mannheim, Germany). Factor V Leiden and FII 20210 GA mutations were determined as described earlier [40]. Total α2-PI antigen and NPB-α2-PI antigen levels were determined by in-house ELISA methods as described earlier [35].

### 4.5. Statistical Analysis

The distribution of the data was tested by the Kolmogorov–Smirnov test with Lilliefors correction. Normally distributed data are presented as mean ± standard deviation (SD), whereas non-normally distributed data are presented as median and interquartile range (IQR). According to a Sample Size Calculator, our sample size (200 cases/group) enables us to detect a 3.3% difference in mean values of β-AT activity with α = 0.05 and β = 0.8. To determine the correlation between continuous variables, Spearman’s correlation coefficient was used. According to a Sample Size Calculator, our sample size (200 cases/group) enables the detection of an r = 0.28 correlation coefficient with α = 0.05 and β = 0.8. To determine parameters showing independent association with the AT levels, multiple linear regression analyses were performed, where fibrinogen, total cholesterol, BMI, hypertension, age, CRP, gender, and total AT (for β-AT) were included as predictors. Differences in category frequencies were evaluated by the Chi-square test. The differences in total AT activity, β-AT activity, and the ratio of β-AT within total-AT were assessed with Mann–Whitney U-test. To study the effect of total-AT activity levels, β-AT activity levels, and the ratio of β-AT within total AT (%) on the risk of VTE, cases were categorized according to the 33.3rd and 66.7th percentile levels of the control group, and cases with AT levels in the lower third were compared to cases with AT levels in the upper third. Odds ratios (ORs) and 95% confidence intervals (95% CIs) were calculated. Adjusted ORs were obtained by using a logistic regression model that included the investigated parameter and other parameters independently associated with the risk of VTE. BMI and CRP were included as continuous variables after natural log-transformation. A *p*-value of <0.05 was considered statistically significant. All statistical analyses were performed using the Statistical Package for Social Sciences software package (SPSS version 29.0 for Macintosh, Chicago, IL, USA).

## 5. Conclusions

β-AT activity levels and the ratio of β-AT within total AT (%) were significantly elevated, while total AT activity did not change in patients with VTE. The increased β-AT activity levels and the ratio of β-AT within total AT (%) were independently associated with VTE risk.

## Figures and Tables

**Table 1:** Clinical and laboratory variables of controls and patients with VTE.

	Control Group (*n* = 200)	VTE Group (*n* = 200)	Significance (*p*)
Age (mean ± SD)	41.1 ± 13.1	41.5 ± 13.4	0.540
Gender (male/female ratio); *n*	89/111	101/99	0.271
Positive family history; *n* (%)	60 (36.1)	61 (34.3)	0.736
Smoking; *n* (%)	40 (20.3)	48 (24.0)	0.399
High blood pressure; *n* (%)	40 (20.1)	97 (48.5)	<0.001
Diabetes mellitus; *n* (%)	0	24 (12.0)	-
Factor V Leiden heterozygotes; *n* (%)	17 (12.5)	62 (31.2)	<0.001
Factor V Leiden homozygotes; *n* (%)	0	11 (5.5)	<0.001
FII 20210G>A carriers; *n* (%)	5 (3.7)	12 (6.0)	0.449
Type of VTE			
DVT; *n* (%)	0	152 (76.0)	-
DVT + PE; *n* (%)	0	42 (21.0)	-
Distal; *n* (%)	0	88 (55.0)	-
Proximal; *n* (%)	0	66 (41.0)	-
Upper extremity; *n* (%)	0	6 (4.0)	-
PE; *n* (%)	0	6 (3.0)	-
Anticoagulant therapy; *n* (%)			
Vitamin K antagonist	0	158 (79.0)	-
Heparin	0	12 (6.0)	-
DOAC	0	18 (9.0)	-
No therapy		12 (6.0)	
BMI (median (IQR))	25.2 (21.9–28.3)	29.0 (25.7–32.9)	<0.001
Fibrinogen; g/L (mean ± SD)	3.34 ± 0.60	3.65 ± 0.66	<0.001
CRP; mg/L (median (IQR))	1.75 (1.00–3.85)	3.70 (2.33–6.40)	<0.001
Triglyceride; mmol/L (median (IQR))	1.98 (1.44–2.59)	1.56 (1.10–2.19)	<0.001
Total cholesterol; mmol/L (median (IQR))	4.96 (4.41–5.44)	5.19 (4.51–6.01)	0.055
HDL cholesterol; mmol/L (median (IQR))	1.21 (1.03–1.42)	1.19 (0.99–1.45)	0.951
LDL cholesterol; mmol/L (median (IQR))	3.19 (2.66–3.67)	3.30 (2.69–4.01)	0.382
Total α2-PI antigen; mg/L (mean ± SD)	65.5 ± 7.9	72.3 ± 9.1	<0.001
NPB-α2-PI antigen; mg/L (mean ± SD)	23.1 ± 6.8	30.6 ± 7.1	<0.001

VTE: venous thromboembolism; DVT: deep vein thrombosis; PE: pulmonary embolism; DOAC: direct oral anticoagulant; BMI: body mass index; CRP: C-reactive protein; HDL: high-density lipoprotein; LDL: low-density lipoprotein; α2-PI: α2-plasmin inhibitor; NPB-α2-PI: non-plasminogen binding α2-plasmin inhibitor; Normally distributed variables are presented as mean ± SD; non-normally distributed variables are presented as median (IQR). To compare the two groups, Chi-square, independent-samples Mann–Whitney U, and independent-samples *T* tests were used for categorical, non-normally distributed, and normally distributed variables, respectively.

**Table 2:** Total AT activity and β-AT activity levels, and the ratio of β-AT within total AT in the study groups.

Parameter	Group	Total	Men	Women	Significance (*p*)
Total AT activity (%) (median, IQR)	Control	100 (93–109)	100 (94–112)	99 (92–106)	0.223
	VTE	99 (94–109)	98 (92–107)	101 (94–111)	0.098
β-AT activity (%) (median, IQR)	Control	89.3 (84.0–95.0)	89.0 (81.7–94.0)	90.0 (86.0–95.3)	0.219
	VTE	93.3 (90.0–97.3)	93.0 (89–96)	94.0 (90–99)	0.031
Ratio of β-AT within total AT (%) (mean ± SD)	Control	8.86 ± 0.88	8.74 ± 0.99	8.96 ± 0.78	0.077
	VTE	9.34 ± 0.68	9.36 ± 0.68	9.31 ± 0.67	0.582

Values with normal and non-normal distribution are expressed as mean ± SD and median (IQR, interquartile range), respectively. To compare the difference between groups independent-samples Mann–Whitney U or independent-samples *T* tests were used, depending on the distribution of the variables. Significance (*p*) indicates the level of significance obtained when comparing women and men.

**Table 3:** The association of AT activity levels with the risk of VTE.

Parameter (*n*, Upper Third/Lowest Third in Controls vs. Patients)	OR	95% CI	Significance (*p*)
Total AT activity % (52/52 vs. 52/49)	0.942	0.599–1.482	0.797
adjusted	1.509	0.764–2.98	0.236
β-AT activity % (52/56 vs. 80/16)	5.783	3.077–10.867	<0.001
adjusted	12.207	5.248–28.313	<0.001
Ratio of β-AT within total AT (%) (50/50 vs. 89/12)	6.147	3.362–11.240	<0.001
adjusted	6.400	2.971–13.788	<0.001

Cases with AT activity levels in the upper third (cut-points used: >109%, >95% and >9.55% for total AT activity (%), β-AT activity (%), and the ratio of β-AT within total AT (%), respectively) were compared to cases with AT levels in the lower third (cut-points used: <93%, <88% and <8.66% for total AT activity (%), β-AT activity (%), and the ratio of β-AT within total AT (%), respectively). The middle tertile was excluded from the analysis. For adjustment, the following parameters were included in the logistic regression model: hypertension, FV Leiden mutation, FII 20210G>A mutation, NPB-α2-PI antigen, and natural log-transformed BMI and CRP.

## Data Availability

The data presented in this study are available on request from the corresponding author due to privacy restrictions.

## Supplementary Materials

The following supporting information can be downloaded at: https://www.mdpi.com/article/10.3390/ijms262211151/s1.

## Abbreviations

The following abbreviations are used in this manuscript:

AT	Antithrombin
BMI	Body mass index
CI	Confidence interval
CRP	C-reactive protein
CV	Coefficient of variation
DOAC	Direct oral anticoagulant
DVT	Deep vein thrombosis
EDTA	Ethylenediaminetetraacetic acid
FIIa	Activated factor II
FVIIa	Activated factor VII
FIXa	Activated factor IX
FXa	Activated factor X
GAG	Glycosaminoglycan
HDL	High density lipoprotein
OR	Odds ratio
IQR	Interquartile range
LDL	Low density lipoprotein
NPB-α2 PI	Non-plasminogen-binding α2-plasmin-inhibitor
PE	Pulmonary embolism
SD	Standard deviation
SERPIN	Serin protease inhibitor
VTE	Venous thromboembolism
