# Supplementary material for: β-Antithrombin Levels in Patients with Venous Thromboembolism
Source: Int J Mol Sci. 2025 Nov 18;26(22):11151. doi: 10.3390/ijms262211151 (PMC12653743; doi:10.3390/ijms262211151)
Supplement: Supplementary file 1 [file ijms-26-11151-s001.zip › ijms-3943804-supplementary.pdf]

Supplementary Table S1.
The association of AT activity levels with the risk of VTE.
Model 1.

Parameter	Exp(B)	Significance (*p*)	95% CI for Exp(B)
Hypertension	2.674	0.003	1.399-5.111
FV Leiden mutation	4.413	<0.001	2.181-8.927
FII 20210 G>A mutation	1.979	0.339	0.489-8.012
BMI	11.412	0.008	1.896-68.686
CRP	1.475	0.022	1.059-2.056
NPB-α2-PI antigen	1.129	<0.001	1.081-1.180
Total-AT activity	1.012	0.379	0.986-1.038

Model 2.

Parameter	Exp(B)	Significance (*p*)	95% CI for Exp(B)
Hypertension	2.781	0.005	1.369-5.649
FV Leiden mutation	4.210	<0.001	1.953-9.076
FII 20210 G>A mutation	1.648	0.517	0.364-7.457
BMI	15.093	0.007	2.066-110.258
CRP	1.581	0.012	1.105-2.263
NPB-α2-PI antigen	1.109	<0.001	1.060-1.160
β-AT activity	1.150	<0.001	1.097-1.205

Model 3.

Parameter	Exp(B)	Significance (*p*)	95% CI for Exp(B)
Hypertension	2.259	0.020	1.136-4.492
FV Leiden mutation	4.032	<0.001	1.944-8.364
FII 20210 G>A mutation	0.871	0.851	0.207-3.667
BMI	6.968	0.044	1.050-46.257
CRP	1.434	0.043	1.012-2.033
NPB-α2-PI antigen	1.153	<0.001	1.101-1.207
β-AT ratio	2.607	<0.001	1.761-3.858

Logistic regression analyses were performed treating total AT activity (Model 1.), β-AT activity (Model 2.), and β-AT/total AT ratio (Model 3.) as continuous predictors. For adjustment the following parameters were included in the models: hypertension, FV Leiden mutation, FII 20210 G>A mutation, NPB-α2-PI antigen, and natural log-transformed BMI and CRP.
